# The Fumagillin Gene Cluster, an Example of Hundreds of Genes under *veA* Control in *Aspergillus fumigatus*

**DOI:** 10.1371/journal.pone.0077147

**Published:** 2013-10-07

**Authors:** Sourabh Dhingra, Abigail L. Lind, Hsiao-Ching Lin, Yi Tang, Antonis Rokas, Ana M. Calvo

**Affiliations:** 1 Department of Biological Sciences, Northern Illinois University, DeKalb, Illinois, United States of America; 2 Department of Biomedical Informatics, Vanderbilt University Medical Center, Nashville, Tennessee, United States of America; 3 Department of Chemical and Biomolecular Engineering, University of California Los Angeles, Los Angeles, California, United States of America; 4 Department of Chemistry and Biochemistry, University of California Los Angeles, Los Angeles, California, United States of America; 5 Department of Biological Sciences, Vanderbilt University, Nashville, Tennessee, United States of America; University of New South Wales, Australia

## Abstract

*Aspergillus fumigatus* is the causative agent of invasive aspergillosis, leading to infection-related mortality in immunocompromised patients. We previously showed that the conserved and unique-to-fungi *veA* gene affects different cell processes such as morphological development, gliotoxin biosynthesis and protease activity, suggesting a global regulatory effect on the genome of this medically relevant fungus. In this study, RNA sequencing analysis revealed that *veA* controls the expression of hundreds of genes in *A. fumigatus*, including those comprising more than a dozen known secondary metabolite gene clusters. Chemical analysis confirmed that *veA* controls the synthesis of other secondary metabolites in this organism in addition to gliotoxin. Among the secondary metabolite gene clusters regulated by *veA* is the elusive but recently identified gene cluster responsible for the biosynthesis of fumagillin, a meroterpenoid known for its anti-angiogenic activity by binding to human methionine aminopeptidase 2. The fumagillin gene cluster contains a *veA*-dependent regulatory gene, *fumR* (Afu8g00420), encoding a putative C6 type transcription factor. Deletion of *fumR* results in silencing of the gene cluster and elimination of fumagillin biosynthesis. We found expression of *fumR* to also be dependent on *laeA*, a gene encoding another component of the fungal *velvet* complex. The results in this study argue that *veA* is a global regulator of secondary metabolism in *A. fumigatus*, and that *veA* may be a conduit via which chemical development is coupled to morphological development and other cellular processes.

## Introduction

The filamentous fungus *Aspergillus fumigatus* is one of the most common human fungal pathogens found infecting a large population of immunodepressed patients. This group includes individuals with hematological malignancies, those with genetic immunodeficiencies, patients infected with HIV, and cancer patients treated with chemotherapy [1–6]. This immunodepressed population is currently increasing [7] due to the higher number of organ transplants performed, immunosuppressive and myeloablative therapies for autoimmune and neoplastic diseases, and the HIV pandemic [1,7–9]. The mortality rate resulting from *A. fumigatus* infections in immunodepressed patients ranges from 40% to 90% [7,9–12].

In a previous report we demonstrated that the global regulatory *velvet* gene *veA* controls *A. fumigatus* production of conidia [13], the main inoculum during infection [14,15], and production of gliotoxin [13], a compound with immunosuppressive properties [16–28] also found to inhibit phagocytosis in macrophage and to induce apoptosis [29,30].

*veA* orthologs have been identified and characterized in other fungi [31,32] including other *Aspergillus* species, such as *A. flavus* [33–35], *A. parasiticus* [36] and the model filamentous fungus *A. nidulans* [37]. These previous studies provided abundant evidence of the role of *veA* as a regulator of both fungal morphological development and secondary metabolism. In 2003 our group described for the first time the role of *veA* as a global regulator of secondary metabolism in *A. nidulans*, including production of the mycotoxin sterigmatocystin [37]. *veA* also regulates the biosynthesis of other mycotoxins, including aflatoxin, cyclopiazonic acid and aflatrem in *Aspergillus flavus* [33], the synthesis of trichothecenes in *F. graminearum* [38], and the production of fumonisins and fusarins in *Fusarium* spp, specifically *F. verticillioides* and *F. fujikuroi* [32,39,40]. However, *veA* also controls the synthesis of other secondary metabolites known for their beneficial medical applications, for example, the beta-lactam antibiotic penicillin in *A. nidulans* and *P. chrysogenum* [37,41] as well as cephalosporin C in *Acremonium chrysogenum* [42].

In-depth studies of *A. nidulans veA* and its gene product also revealed mechanistic details of its mode of action. For instance, it is known that the VeA protein is transported to the nucleus by the KapA α-importin, and that this transport is promoted in the absence of light [43,44]. In the nucleus, VeA interacts with light-sensing proteins that also affect secondary metabolism and fungal differentiation, such as the red phytochrome-like protein FphA, which interacts with the blue- responsive proteins LreA-LreB [31,45]. In the nucleus VeA also interacts with VelB and LaeA [46,47]. VelB is another protein in the *velvet* family [47], and LaeA is a chromatin modifying protein that, like VeA, is required for the synthesis of numerous secondary metabolites [48,49]. In addition, a LaeA-like putative methyltransferase was also described to interact with VeA [50].

A microarray-based transcriptome study showed that *A. fumigatus laeA* affects the expression of 13 secondary metabolite gene clusters [51]; however, at that time the extent of *veA* regulation of the activation of secondary metabolite gene clusters was mostly unknown. With the goal of elucidating the full extent of *veA*-regulation of the *A. fumigatus* genome, particularly with respect to genes involved in secondary metabolism, we performed RNA sequencing analyses [52] and chemical characterization of *A. fumigatus* cultures, obtaining results consistent with a global regulatory pattern. This study also contributes to uncovering the regulation of novel secondary metabolite gene clusters in *A. fumigatus*. For example, an important discovery in our study is that *veA* and *laeA*, both of which encode *velvet* complex components, regulate the recently discovered gene cluster responsible for the synthesis of fumagillin [53]. Fumagillin has been intensely studied due to its potential in the treatment of amebiasis [54], microsporidiosis [55] and most recently, for its anti-angiogenic activity as inhibitor of the human type 2 methionine aminopeptidase (MetAP2) [56,57].

## Materials and Methods

### Strains and culture conditions

*Aspegillus fumigatus* strains used in this study are listed in Table S1. Fungal strains were grown on Czapek Dox media (Difco), unless otherwise indicated, and supplements for the corresponding auxotrophies as needed [58]. Solid medium was prepared by adding 15 g/liter agar. Strains were stored as 30% glycerol stocks at -80°C.

### RNA extraction

Total RNA was extracted as previously described [13]. Briefly, conidia from the wild type, deletion *veA* (∆*veA*), complementation and over-expression *veA* (OE*veA*) strains were inoculated in Czapek-Dox (approximately 10^7^ spores/mL) and grown as liquid stationary cultures at 37°C in the dark. Mycelia were collected 48 h and 72 h after inoculation and RNA was extracted using TRIzol (Invitrogen) following the manufacturer’s instructions. RNA samples were further purified using QIAgen RNeasy mini kit as previously described [59].

### Transcriptome analysis

#### Genome and transcriptome sequence versions

All *A. fumigatus* Af293 sequences used are from sequence version s03-m04-v01 from the Aspergillus Genome Database (AspGD) [60].

#### Library preparation and RNA sequencing

RNA-Seq libraries were constructed and sequenced at the Vanderbilt Genome Sciences Resource using the Illumina Tru-seq RNA sample prep kit as previously described [61,62]. In brief, total RNA quality was assessed via Bioanalyzer (Agilent). Upon passing quality control, poly-A RNA was purified from total RNA and the second strand cDNA was synthesized from mRNA. cDNA ends were then blunt repaired and given an adenylated 3’ end. Next, barcoded adapters were ligated to the adenylated ends and the libraries were PCR enriched, quantified, pooled and sequenced an on Illumina HiSeq 2000 sequencer.

#### Read alignment and quantification of gene expression

Illumina TruSeq adapters were trimmed from the 3’ end of reads using the scythe software package (available from Buffalo, V. at https://github.com/ucdavis-bioinformatics/scythe), and low-quality bases were trimmed using the sickle software package (available from Joshi, N. at https://github.com/ucdavis-bioinformatics/sickle). Reads were aligned to the transcriptome using the bowtie read alignment software for single-end reads, with the maximum mismatches per read set at 2 and a seed length of 28 [63]. Read count per gene was calculated using SAMtools idxstats software [64]. For each sample, gene expression was quantified using the reads per Kilobase of exon per million mapped reads (RPKM) metric [65]. Differential expression was calculated between ∆*veA* and wild type, between OE*veA* and wild type, and between the complementation and wild-type strains. Two cutoffs were used to determine differentially regulated genes [61,62]. The first cutoff compared the fold difference between genes by calculating the relative RPKM (rRPKM = RPKM_sample1_/RPKM_sample2_) for each gene. The second cutoff compared the proportion of reads mapping to a gene in different samples using Fisher’s exact test with Bonferroni’s correction for multiple comparisons. A gene was considered differentially regulated if the log_2_ rRPKM value was equal to or greater than 2 and the Bonferroni-corrected Fisher’s exact *p*-value was less than 0.05.

#### Gene ontology categorization

The gene ontology (GO) categorizations of differentially regulated genes were compared against the set of non-differentially regulated genes to identify GO categories that were specifically enriched in differentially upregulated or downregulated gene sets in the three strain comparisons. GO categorizations for each gene were obtained from the AspGD’s GOSlim mapper for *A. fumigatus* Af293 [60]. AspGD’s GOSlim mapper contains higher-order GO terms for the process, component, and function sections of GO. All comparisons were performed using Fisher’s exact test with Bonferroni’s correction for multiple comparisons.

#### Sliding window analysis

We used a sliding window analysis [61] to determine clusters of genes that were upregulated or downregulated in the *A. fumigatus* genome in ∆*veA* versus wild type and OE*veA* versus wild type comparisons. Briefly, each gene was encoded as upregulated, downregulated or not significantly differentially regulated according to our specified differential regulation cutoffs. Then, we calculated the cumulative binomial probability of observing every window of 24 genes along each chromosome. To account for multiple comparisons, we used an empirically derived false discovery rate (FDR) by randomly permuting the expression data 1,000 times and running the sliding window analysis on each permuted dataset. Our FDR cutoff was set conservatively at 0.01. After all clusters below this FDR cutoff were found, genomically overlapping windows were collapsed into a larger cluster. Due to the window size, regions at the beginning or end of these master clusters may contain stretches of non-differentially regulated genes. We have reported all clusters from the first to last differentially regulated gene found for each cluster and the start and end genes of all significant clusters.

### Quantitative RT-PCR analysis

One microgram of total RNA was treated with RQI Dnase to remove possible DNA contamination. Then cDNAs were obtained by reverse transcription using Moloney murine leukemia virus (MMLV) reverse transcriptase (Promega). Quantitative Real Time PCR was performed with an Agilent MX3000p thermocycler using SYBR green Jump Start *Taq* (Sigma). The primers used for gene expression analysis are listed on Table S2.

### Metabolome analysis

#### Extractions of secondary metabolites

Secondary metabolites were extracted as previously described [13]. Briefly, liquid Czapek-dox stationary cultures of the wild type, ∆*veA*, complementation and OE*veA* strains were grown as described. Supernatants were collected by filtration through sterile Miracloth™ (Calbiochem, USA) from 72 h and 120 h cultures. Fifteen mL of the culture filtrate was extracted with same amount of chloroform. Extracts were allowed to dry and were resuspended in 500µL of methanol; 10 µL aliquots were used for LC-MS analysis.

#### LC-MS

All solvents and other chemicals used were of analytical grade. All LC-MS analyses were performed on a Shimadzu 2010 EV LC-MS (Phenomenex® Luna, 5μ, 2.0 × 100 mm, C18 column) using positive and negative mode electrospray ionization with a linear gradient of 5–95% MeCN-H_2_O (0.1% formic acid) in 30 minutes followed by 95% MeCN for 15 minutes with a flow rate of 0.1 mL/min. The value of area under curve was observed by EIC (extracted ion chromatogram).

### Generation of the *fumR* (Afu8g00420) deletion strain

Fusion Polymerase Chain Reaction (Fusion PCR) was used to create the deletion cassette of *fumR* as previously described [66]. First, *Aspergillus parasiticus pyrG* was PCR amplified from *A. parasiticus* genomic DNA using primers AparapyrGF-Linker and AparapyrGR-Linker (Table S2). The *Aspergillus parasiticus pyrG* fragment was ligated into pJET (Fermentas) yielding plasmid pSD38.1 Then, 1.5kb of 5’ UTR and 3’ UTR of *fumR* was amplified from *A. fumigatus* genomic DNA using primer pairs 420P1 & 420P2 and 420P3 & 420P4, respectively (Table S2). *Aspergillus parasiticus pyrG* was then amplified from pSD38.1 using primers 420P5 and 420P6. Three fragments were fused using primers 420P7 and 420P8 (Table S2). Protoplast mediated fungal transformation was done as previously described [66] using CEA17ku80 (gift from Robert Cramer) as the host strain*. Aspergillus parasiticus pyrG* was utilized as selectable marker, resulting in a complete gene replacement of *fumR* in CEA17ku80. Transformants were first screened using PCR (data not shown) and Southern blot analysis. Other DNA manipulations were done as previously described [67].

### Generation of the *laeA* deletion strain

The *laeA* deletion DNA cassette was also generated by fusion PCR. Briefly, a 1.5 kb 5’ UTR fragment was first amplified from *A. fumigatus* genomic DNA with primers laeA_p1 and laeA_p2 (Table S2). A 1.3 kb 3’ UTR fragment was also amplified from genomic DNA with primers laeA_p3 and laeA_p4 (Table S2). *Aspergillus parasiticus pyrG* was amplified from pSD38.1 using primers laeA_p5 and lae_p6. The three fragments were fused using primers laeA_p7 and laeA_p8 (Table S2) as previously described [66]. The *laeA* deletion cassette was transformed into CEA17ku80. Transformants were first screened using PCR (data not shown) and Southern blot analysis.

## Results

### Hundreds of genes are differentially regulated by *veA*

**Figure 1:**
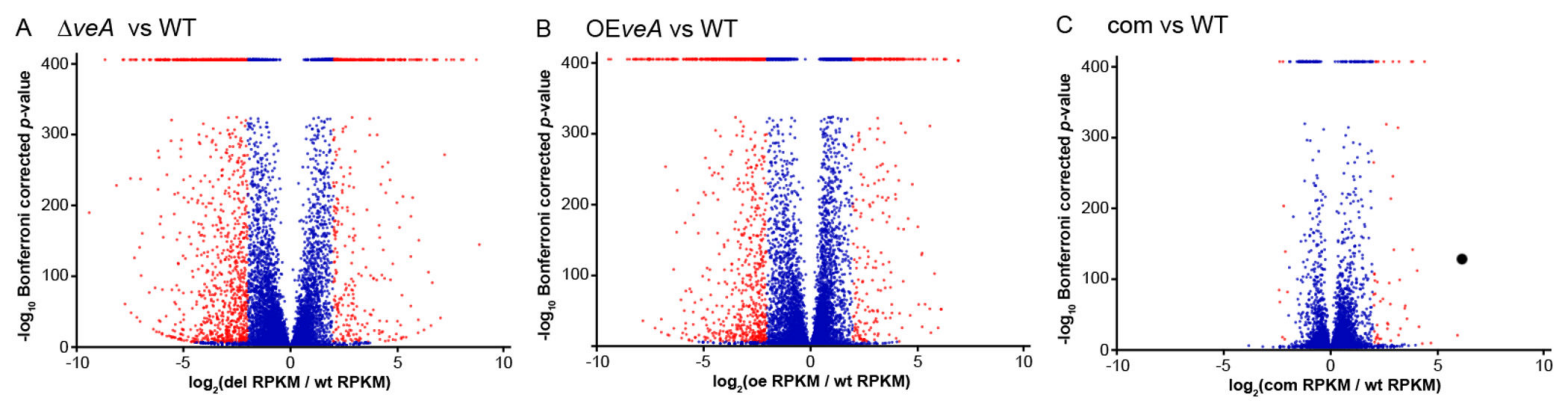
Differentially regulated genes in comparisons of *veA* deletion, overexpression and complementation strains against the wild-type strain. For each gene, the log_2_ rRPKM is reported along the x-axis and the negative log_10_ of the Bonferroni-corrected Fisher’s Exact *p*-value is reported along the y-axis. *P*-values with an infinite log_10_ value (*p*-values equal to zero) are reported as 400. Genes with a log_2_ rRPKM greater than 2 and a Bonferroni-corrected *p*-value less than 0.05 are considered differentially regulated and are represented by red dots, whereas genes below these thresholds are not considered to be differentially regulated and are represented by blue dots. (A) Comparison between the strain that carries a deletion of the *veA* gene (∆*veA*) and the wild-type (WT) strain; (B) Comparison between the *veA* over-expression strain (OE*veA*) and the WT strain; (C) Comparison between the *veA* complementation strain (com) and the WT strain.

Of the 9,784 genes in the *A. fumigatus* genome [60,68], 453 were upregulated and 1,137 were downregulated in the ∆*veA* strain when compared with the wild-type strain (Figure 1; Table S3). A similar pattern was observed in the OE*veA* versus wild type comparison, where 335 genes were upregulated and 908 genes were downregulated in the OE*veA* strain. In sharp contrast, comparison of the complementation strain and wild type showed that the two strains present very similar expression patterns.

### Differentially regulated genes are dramatically enriched for secondary metabolism-related processes

Different GO process, component, and function categories are significantly enriched for both upregulated and downregulated genes in the two comparisons (Table 1). Importantly, enrichment analysis using GOSlim categories showed that differentially regulated genes in the ∆*veA* versus wild type and OE*veA* versus wild type comparisons have significant functional overlap (Table 1); specifically, 13 of the 19 GO categories that are enriched for either upregulated (3 categories) or downregulated (16 categories) genes in the ∆*veA* versus wild type comparison are also enriched and in the same direction in the OE*veA* versus wild type comparison. For example, both secondary metabolic process (GO:0019748) and toxin metabolic process (GO:0009404) GO function categories are enriched in downregulated genes from both comparisons.

**Table 1:** The List of GOSlim Categories That Are Significantly Enriched For Differentially Expressed Genes Between The *veA* Deletion (Del) And The Wild-Type (WT) Strain As Well As Between The *veA* Overexpression (OE) And The WT Strain.

**Comparison**	**GO Category ID**	**GO Category Description**	***p*-value**
***GO Process Categories that are Significantly Enriched for Upregulated Genes***			
Del vs WT	GO:0042254	ribosome biogenesis	1.12E-06
.	GO:0016070	RNA metabolic process	3.33E-02
OE vs WT	GO:0005975	carbohydrate metabolic process	1.03E-02
.	GO:0016070	RNA metabolic process	1.86E-02
***GO Process Categories that are Significantly Enriched for Downregulated Genes***			
Del vs WT	GO:0019748	secondary metabolic process	2.61E-34
.	GO:0006996	organelle organization	1.22E-13
.	GO:0016070	RNA metabolic process	1.24E-10
.	GO:0009404	toxin metabolic process	1.38E-09
.	GO:0006464	cellular protein modification process	3.31E-05
.	GO:0042254	ribosome biogenesis	7.55E-05
.	GO:0006259	DNA metabolic process	1.93E-04
.	GO:0006810	transport	2.16E-04
.	GO:0007049	cell cycle	5.40E-03
.	GO:0005975	carbohydrate metabolic process	9.41E-03
.	GO:0016192	vesicle-mediated transport	3.88E-02
OE vs WT	GO:0019748	secondary metabolic process	5.21E-44
.	GO:0006996	organelle organization	1.98E-13
.	GO:0009404	toxin metabolic process	1.30E-09
.	GO:0016070	RNA metabolic process	5.18E-06
.	GO:0006810	transport	1.31E-05
.	GO:0042254	ribosome biogenesis	1.20E-03
.	GO:0006464	cellular protein modification process	1.38E-03
.	GO:0007049	cell cycle	4.27E-03
.	GO:0006259	DNA metabolic process	1.92E-02
.	GO:0016192	vesicle-mediated transport	4.35E-02
.	GO:0006950	response to stress	4.99E-02
***GO Component Categories that are Significantly Enriched for Upregulated Genes***			
Del vs WT	GO:0005730	nucleolus	4.57E-05
OE vs WT	GO:0005886	plasma membrane	1.46E-04
.	GO:0005576	extracellular region	1.67E-04
.	GO:0005618	cell wall	1.72E-03
***GO Component Categories that are Significantly Enriched for Downregulated Genes***			
Del vs WT	GO:0005576	extracellular region	4.19E-18
.	GO:0005634	nucleus	8.60E-06
OE vs WT	GO:0005576	extracellular region	2.14E-07
***GO Function Categories that are Significantly Enriched for Upregulated Genes***			
OE vs WT	GO:0005215	transporter activity	4.01E-02
***GO Function Categories that are Significantly Enriched for Downregulated Genes***			
Del vs WT	GO:0016491	oxidoreductase activity	1.56E-06
.	GO:0005198	structural molecule activity	1.32E-02
.	GO:0005515	protein binding	3.63E-02
OE vs WT	GO:0016491	oxidoreductase activity	5.25E-06

### Differentially regulated genes are non-randomly distributed across the *A. fumigatus* genome

**Figure 2:**
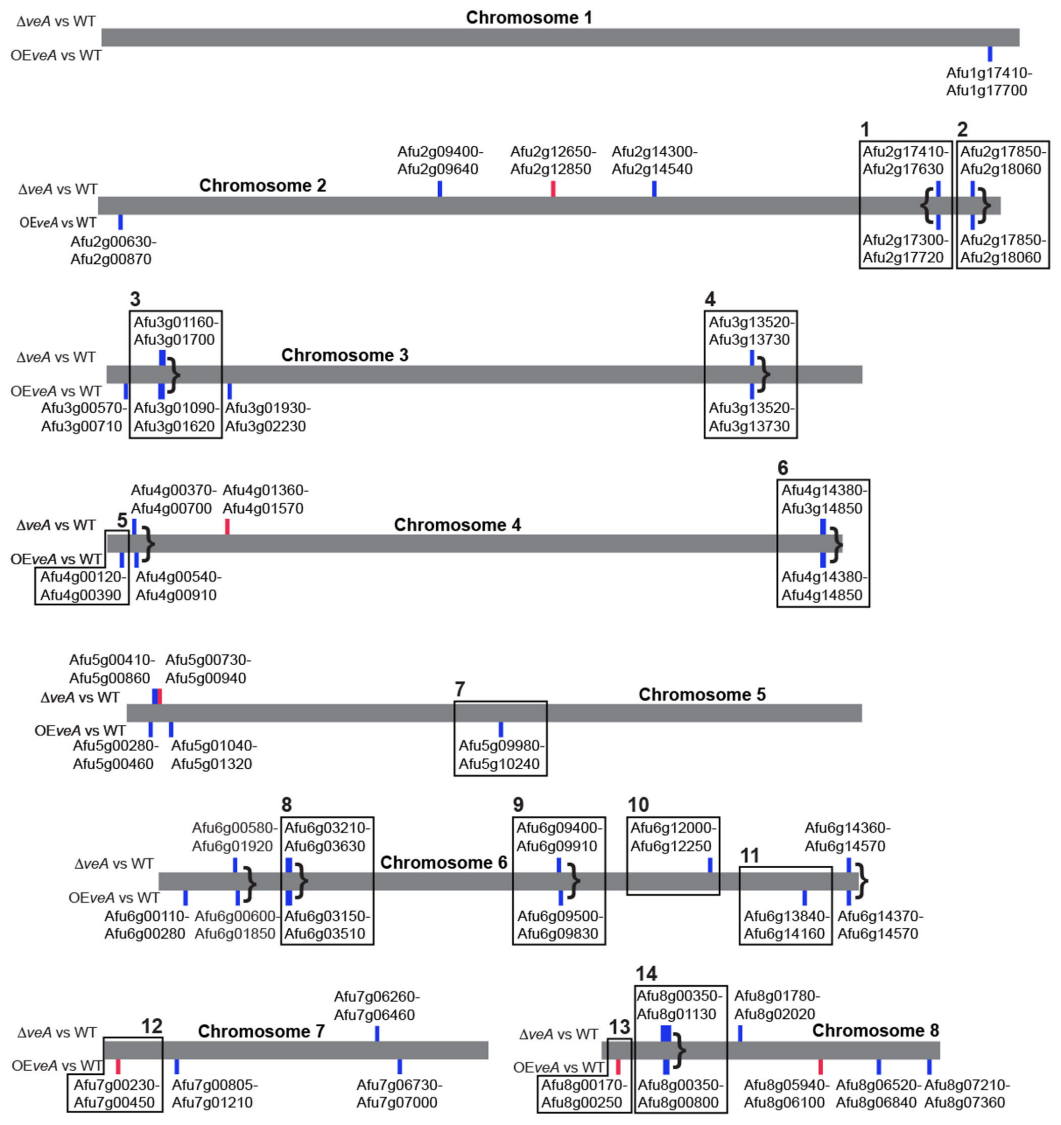
The genome-wide distribution of *de novo* identified gene clusters that appear to be regulated by *veA*. De novo identification of gene clusters differentially regulated by the deletion versus wild-type (shown above each chromosome) and overexpression versus wild-type (shown below each chromosome) comparisons. Upregulated gene clusters are represented by red boxes and downregulated gene clusters are represented by blue boxes. Gene clusters identified in both comparisons whose boundaries overlap are denoted by bracket symbols. Previously reported secondary metabolism gene clusters [60,71] are boxed and numbered. 1: This cluster contains a conidial pigment biosynthesis cluster from Afu2g17530 – Afu2g17600 [82]; 2. This cluster contains the Fumigaclavine C cluster from Afu2g17960 – Afu2g18060 [83]; 3. Cluster of unknown function; 4. Cluster of unknown function; 5. This cluster contains an endocrocin secondary metabolism cluster from Afu4g00210 – Afu4g00230 [84]; 6. This cluster contains the pathway responsible for helvolic acid biosynthesis from Afu4g14770 – Afu4g14850 [85]; 7. Cluster of unknown function; 8. Cluster of unknown function; 9. This cluster contains the gliotoxin cluster from Afu6g09630 – Afu6g 09740 [86]; 10. This cluster contains the fumiquinazoline biosynthetic cluster from Afu6g12040 – Afu6g12110 [87]; 11. Cluster of unknown function; 12. Cluster of unknown function; 13. This cluster contains the fumitremorgin G cluster from Afu8g00260 – Afu8g00170 [76]; 14. This cluster contains the fumagillin gene cluster from Afu8g00370 – Afu8g00520 [53]; and the pseurotin A cluster from Afu8g00530 – Afu8g00570 [88].

**Figure 3:**
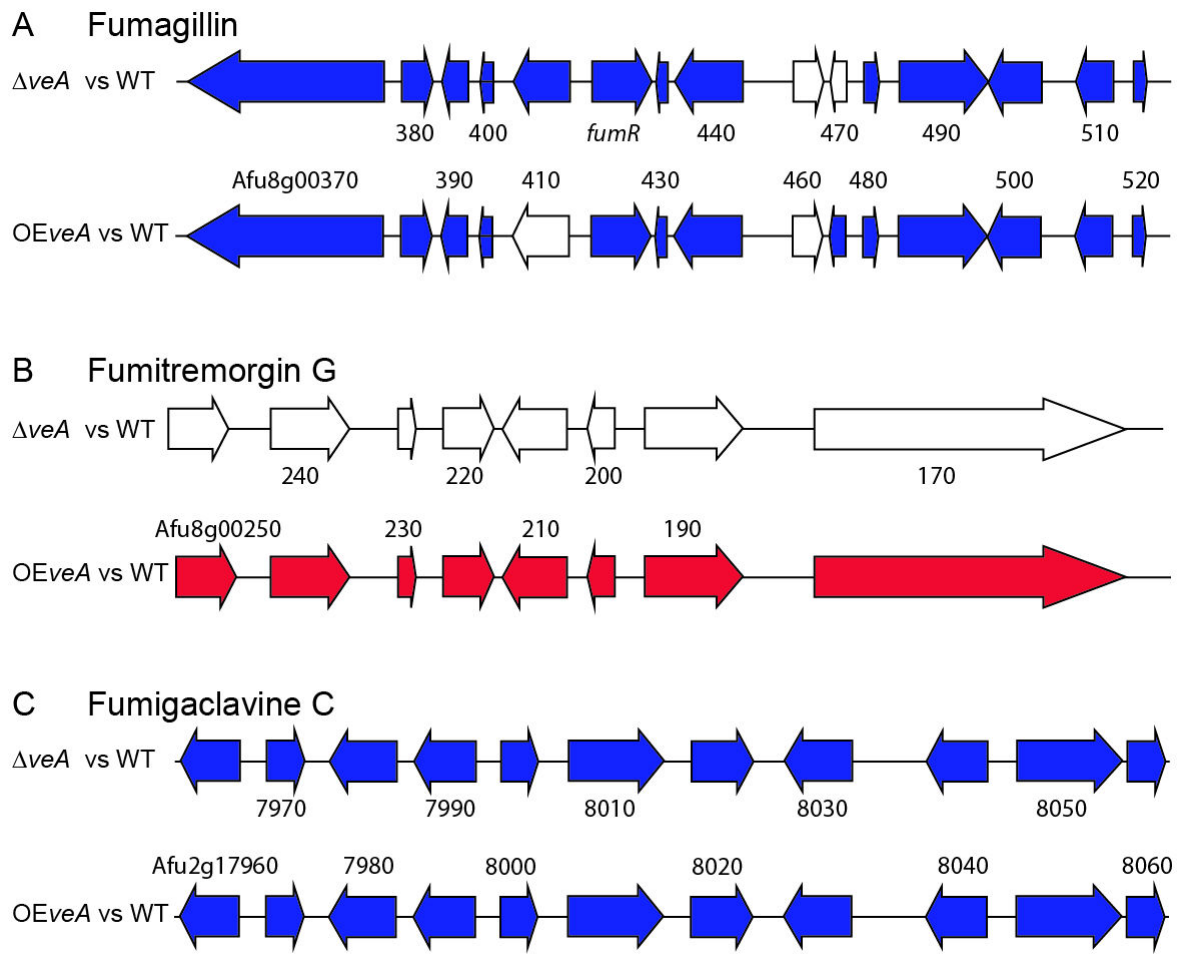
Expression patterns of the fumagillin (A), fumitremorgin G (B), and fumigaclavine C (C) gene clusters in the Δ*veA* vs WT and OE*veA* vs WT comparisons. Genes labeled with numbers correspond to locus tags without the chromosomal prefix or trailing zeros (i.e., the gene labeled 380 in the fumagillin cluster corresponds to Afu8g00380). The sole exception is *fumR* (Afu8g00420), the name given to the regulatory gene for the fumagillin cluster characterized in this study. Genes are color-coded by differential expression; blue indicates downregulation, red indicates upregulation, and white indicates no differential regulation.

We used a sliding window analysis [2] to determine clusters of genes that were upregulated or downregulated for the ∆*veA* versus wild type and OE*veA* versus wild type comparisons. In total, 31 downregulated gene clusters and 6 upregulated gene clusters were identified (Figure 2). Ten downregulated clusters were found independently in both the ∆*veA* versus wild type and OE*veA* versus wild type comparisons, suggesting some similarity in phenotype between the α*veA* and OE*veA* strains. Twelve of the 31 downregulated clusters and 2 of the 6 upregulated clusters overlap with known or predicted secondary metabolic gene clusters [1,9]. For example, the gene clusters encoding for the secondary metabolites fumagillin, fumitremorgin G, and fumigaclavine C are all differentially regulated in at least one of the two strain comparisons (Figure 3). All 11 genes in the fumigaclavine C biosynthetic gene cluster are downregulated in both the ∆*veA* versus wild type and OE*veA* versus wild type comparisons. In both the ∆*veA* versus wild type and OE*veA* versus wild type comparisons, 13 of the 15 genes in the fumagillin gene cluster are downregulated. All genes involved in fumitremorgin G are upregulated on the OE*veA* versus wild type comparison, but none of these genes are differentially regulated in the α*veA* versus wild type comparison.

### *veA* regulation profile of secondary metabolite gene clusters in *A. fumigatus* partially differs from the *laeA* profile

Previous studies of the model fungus *Aspergillus nidulans* demonstrated that the *veA* gene product, VeA, interacts with other proteins in cell nuclei [45,47], among them LaeA. Additionally, Park et al. [69] used tandem affinity techniques [47] to show that the *A. fumigatus* LaeA co-purifies with *A. nidulans* VeA, suggesting that *A. fumigatus* VeA might also interact with *A. fumigatus* LaeA. LaeA is a putative methyl transferase that affects chromatin conformation [48]. This VeA-interacting protein has also been described as affecting expression of secondary metabolite gene clusters in *A. fumigatus* [51]. Our results indicate that although the regulation patterns of the two proteins overlap, they are not identical (Table 2). Specifically, five out of the nine clusters that Perrin et al. report as being under full *laeA* regulation are found to be in windows of differentially regulated genes in both the ∆*veA* versus wild type and the OE*veA* versus wild type comparisons. Of the remaining four clusters, two are not found to be differentially regulated and two have different expression patterns in the two comparisons. The Afu6g12040–2080 *laeA*-regulated cluster was found to be part of a window of downregulated genes in the ∆*veA* versus wild type comparison but not in the OE*veA* versus wild type comparison. However, all five genes in this cluster are also downregulated in the OE*veA* versus wild-type comparison, and its lack of detection by the sliding window analysis is due to the gene cluster’s small size and the test’s stringency. Finally, of the four clusters Perrin et al. describe as being partially regulated by *laeA*, two are differentially regulated in both *veA* comparisons, one cluster is differentially expressed in the OE*veA* versus wild type but not in the ∆*veA* versus wild type comparison, and one cluster is not differentially expressed in either comparison.

**Table 2:** The correspondence between gene clusters defined in the studies by Perrin et al. and Inglis et al. with our sliding window analysis.

Gene cluster borders defined by Perrin et al.	Gene cluster name defined by Inglis et al.	Gene cluster borders defined by SMURF analysis (Inglis et al.)	Gene cluster borders defined by antiSMASH analysis (Inglis et al.)	Gene cluster borders defined by Inglis et al.	**Window of genes downregulated in** α***veA* vs WT comparison**	**Window of genes downregulated in OE*veA* vs WT comparison**	Additional notes
**Full LaeA regulation**							
Afu1g10360–0390	Afu1g10380 (nrps1) cluster	Afu1g10310–0380	Afu1g10310–0420	Afu1g10270–0380	None	None	
Afu2g17510–7600	Afu2g17600 cluster	Afu2g17511–7600	Afu2g17490–7690	Afu2g17480–7600	Afu2g17410–7630	Afu2g17300–7720	conidial pigment biosynthesis cluster (Afu2g17530–7600)
Afu2g17960–8070	Fumigaclavine C (fga) cluster	Afu2g17930–8070	Afu2g17950–8070	Afu2g17960–8060	Afu2g17850–8060	Afu2g17850–A8060	
Afu3g12870–3010	Afu3g12920 cluster	Afu3g12960–2750	Afu3g13020–2820	Afu3g12960–2890	None	None	
	Afu3g12930 cluster	Afu3g13000–2750	Afu3g13020–2820	Afu3g12960–2890			
Afu6g03290–3490	Afu6g03480 cluster	Afu6g03620–3430	Afu6g03550–3400	Afu6g03490–3430	Afu6g03210–3630	Afu6g03150–3510	
Afu6g09580–9770	Afu6g08560 cluster	Afu6g08540–8560	Afu6g08520–8640	Afu6g08550–8560	Afu6g09400–9910	Afu6g09500–9830	
Afu6g12040–2080	Afu6g12080 cluster	Afu6g12040–2160	Afu6g11980–2145	Afu6g12040–2080	Afu6g12000–2250	None	fumiquinazoline biosynthetic cluster (Afu6g12040–2110); all genes between Afu6g12030–2090 in the OE*veA* vs WT comparison are downregulated
Afu6g13920–4000	Afu6g13930 cluster	Afu6g13830–4050	Afu6g13820–4030	Afu6g13920–4000	None	Afu6g13840–4160	
Afu8g00100–0720	Afu8g00540 cluster	Afu8g00370–0370	Afu8g00490–0310	None	Afu8g00350–1130	Afu8g00350–0800	Afu8g00170–0250 is upregulated in OE*veA* vs WT comparison; fumitremorgin G (ftm) cluster (Afu8g00260–0170); fumagillin gene cluster (Afu8g00370–0520); pseurotin A cluster (Afu8g00530–0570)
	Afu8g00620 cluster	Afu8g00640–0470	Afu8g00720–0390	None			
**partial LaeA regulation**							
Afu3g01290–1600	Afu3g01410 cluster	Afu3g01400–1560	Afu3g01360–1560	Afu3g01400–1480	Afu3g01160–1700	Afu3g00570–0710	
Afu3g14560–4760	Afu3g14700 cluster	Afu3g14880–4690	Afu3g14820–4620	Afu3g14730–4690	None	None	
Afu4g00110–0280	Afu4g00210 cluster	Afu4g00260–0210	Afu4g00290–0150	Afu4g00260–0200	None	Afu4g00120–0390	endocrocin secondary metabolism cluster (Afu4g00210–0230)
Afu4g14380–4850	Afu4g14560 cluster	Afu4g14730–4420	Afu4g14660–4440	Afu4g14610–4450	Afu4g14380–4850	Afu4g14380–4850	helvolic acid biosynthesis (Afu4g14770- 4850)

### *veA* regulates the synthesis of fumagillin, fumitremorgin G, fumigaclavine C and glionitrin A

**Figure 4:**
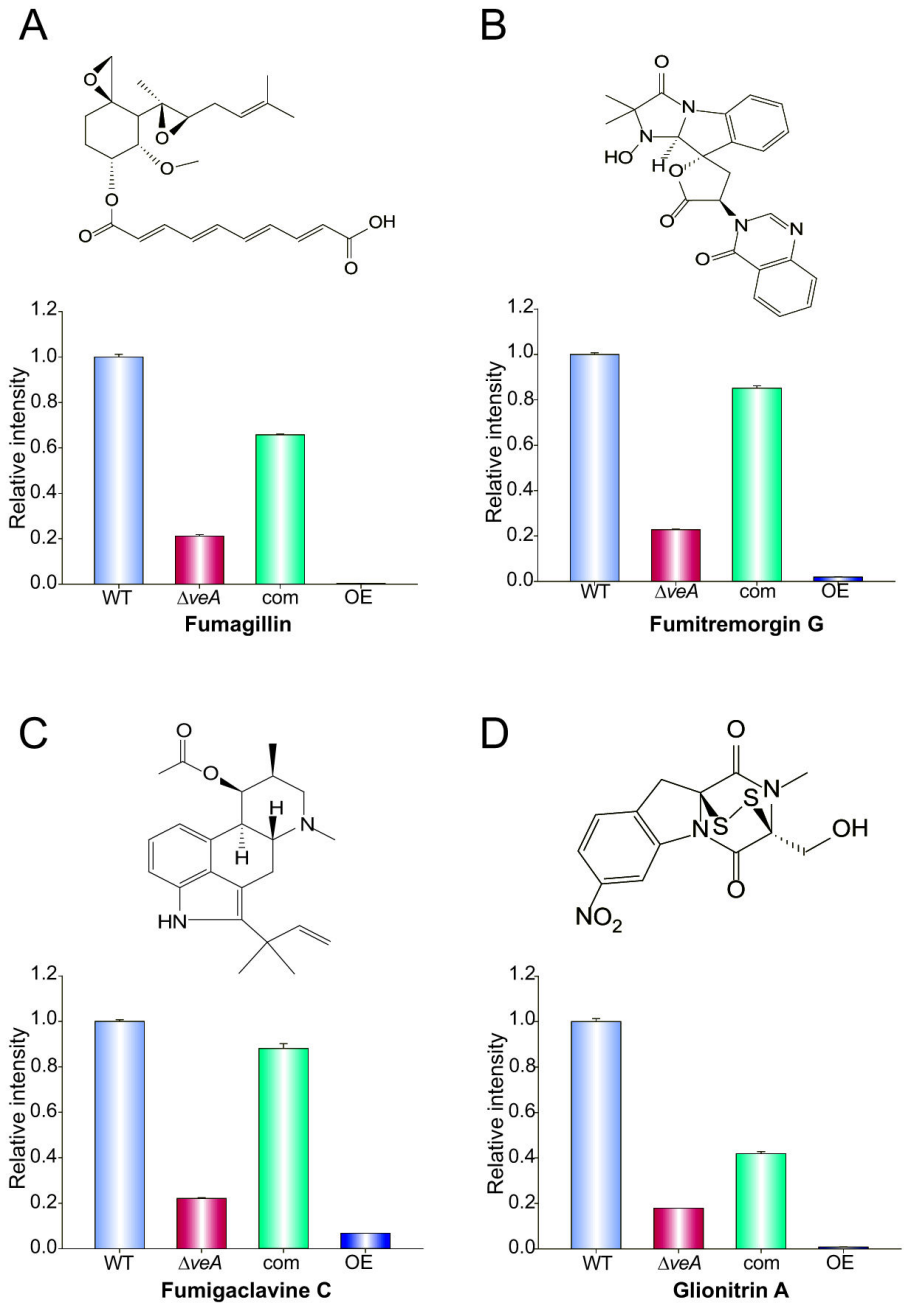
*veA* regulates the production of fumagillin, fumitremorgin G, fumigaclavine C and Glionitrin A. Secondary metabolites were extracted from 120 h old Czapek-dox stationary liquid cultures of wild type (WT), Δ*veA*, complementation and OE*veA* strains. Extracts were analyzed with Shimadzu 2010 EV LC-MS as described in the materials and methods section. The predicted m/z [M+H]^+^ ratio was (A) fumagillin (m/z=459), (B) fumitremorgin G (m/z = 433) (C) fumigaclavine C (m/z = 299) and (D) Glionitrin-A (m/z = 354). The bars represent the mean of three samples and error bars represent standard error.

*Aspergillus fumigatus* has the potential to produce 226 bioactive secondary metabolites [70], and the genes responsible for their synthesis are commonly associated in the form of gene clusters. Recently, Inglis et al. described 39 secondary metabolite gene clusters in the *A. fumigatus* genome [71], some of which are experimentally characterized and some of which are computationally predicted. The production of a number of secondary metabolites has been shown to be under the control of *veA* orthologs in different fungal species [32,33,37–42]. In *A. fumigatus* we recently reported that the expression of gliotoxin genes and gliotoxin production are regulated by *veA* [13]. Additionally, in our current study, our RNA-seq data indicates that the expression of many secondary metabolite gene clusters is also *veA*-dependent, strongly suggesting that *veA* affects the synthesis of other natural products in *A. fumigatus*. For this reason, we also used LC-MS to analyze the production of other compounds in wild type, ∆*veA*, complementation strain and OE*veA* cultures. Our data revealed that production of four additional secondary metabolites was also dependent on *veA* under the experimental conditions assayed, specifically fumagillin, fumitromorgin G, fumigaclavine C and glionitrin A. The production of these four compounds was notably decreased in the ∆*veA* strain. Relative amounts of fumagillin, fumitremorgin G, fumigaclavine C and glionitrinA in the ∆*veA* strain were 18%, 23%, 22% and 18% compared to the wild type levels, respectively (Figure 4). The production of these compounds was also reduced in the OE*veA* with only 0.3%, 0.1%, 0.7% and 0.8% as compared to wild-type levels, respectively (Figure 4).

### *veA* controls the fumagillin gene cluster and fumagillin production by regulating the expression of *fumR* (Afu8g00420)

Due to the medical applications of fumagillin and fumagillin-related compounds for their potential use in the treatment of amebiasis, microsporidiosis, and for their anti-angiogenic properties, we further characterized the genetic regulation of *veA* on the fumagillin gene cluster. RNA-seq analysis revealed that *veA* controls the fumagillin gene cluster, including *fumR* (Afu8g00420) (Figures 2 and 3), a gene encoding a putative C6 type transcription factor that our previous bioinformatics analysis identified within the fumagillin gene cluster, located in chromosome 8 [53]. We further validated these results by qRT-PCR analysis (Figure S1). The expression levels of *fumR* were 12% in ∆*veA* and 5% in OE*veA* with respect to the levels in the wild type strain. Expression of Afu8g00370, which encodes a polyketide synthase (PKS) in the fumagillin cluster [53], was also evaluated (Figure S1). We recently showed that expression of Afu8g00370 is necessary for the production of fumagillin, confirming the predicted role of Afu8g00370 as an indispensable PKS in fumagillin biosynthesis. Our data indicated that expression levels in ∆*veA* and OE*veA* strains were only 3% and 0.1%, respectively, compared to wild-type levels.

### *fumR* is necessary for the expression of other genes in the fumagillin gene cluster

To gain insight into the function of the transcription factor encoded by *fumR*, this gene was deleted by gene replacement techniques using the *A. parasiticus pyrG* marker as described in the materials and methods section. The ∆*fumR* strain was confirmed by PCR (data not shown) and Southern blot analysis (Figure S2). Deletion of *fumR* did not change growth rate or development in *A. fumigatus* (data not shown).

**Figure 5:**
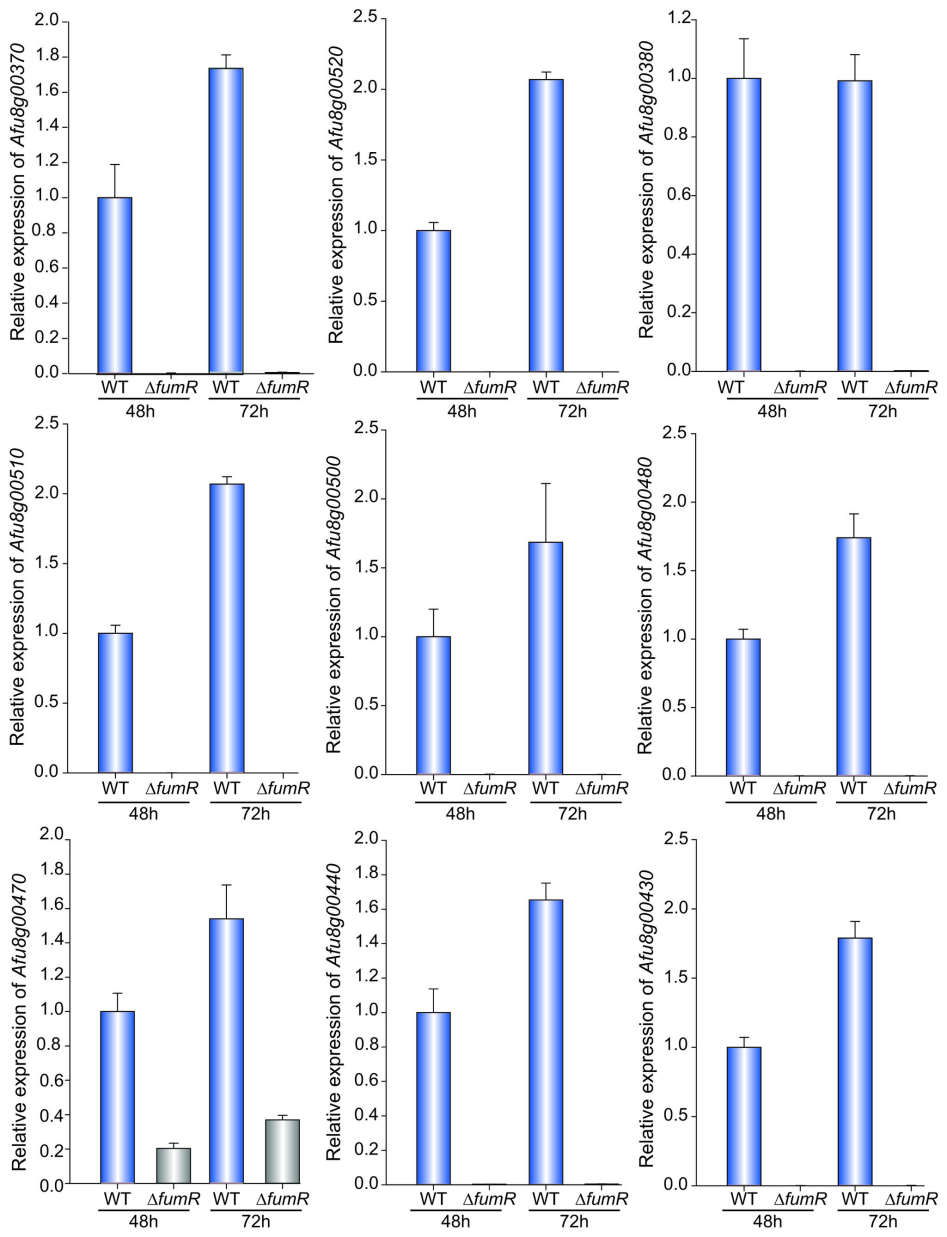
*fumR* is necessary for the expression of the genes in the fumagillin cluster. The transcriptional pattern of the genes in the fumagillin cluster (Afu8g00370, Afu8g00520, Afu8g00370, Afu8g00510, Afu8g00500, Afu8g00480, Afu8g00470, Afu8g00440, Afu8g00430) was evaluated by qRT-PCR in the wild type (WT, TSD51.1) and α*fumR* strains. Total RNA was extracted from Czapek-Dox stationary liquid cultures incubated for 48 h and 72 h. The relative expression was calculated using 2^-ΔΔCT^ as described by Schmittgen and Livak [89]. 18S gene expression was used as internal reference. Means of three replicates is shown. Values were normalized to WT expression at 48 h considered as 1. Error bar represents standard error.

Our experiments showed that expression of the PKS gene, Afu8g00370, is regulated by *fumR* (Figure 5). The expression of Afu8g00370 in the Δ*fumR* strain and control strain was analyzed by qRT-PCR at 48 h and 72 h post inoculation. At both time points examined, there was only negligible expression of Afu8g00370 in the ∆*fumR* mutant as compared to the wild-type levels. Furthermore, our analysis revealed that the expression of other genes in the fumagillin cluster is also under the control of *fumR* (Figure 5). We examined the expression of Afu8g00520, the terpene cyclase involved in fumagillin biosynthesis [53] and Afu8g00380, an essential acyltransferase for fumagillin production [53]. In addition to the characterized genes in the fumagillin cluster, we analyzed the expression of other predicted genes in the cluster, including Afu8g00510, Afu8g00500, Afu8g00480, Afu8g470, Afu8g440 and Afu8g00430. Our results indicate that all genes were minimally expressed in the ∆*fumR* mutant compared to the wild type at both time points tested (Figure 5). Only Afu8g00470 showed slightly higher expression than the other genes in the cluster in ∆*fumR* (20% and 36% at 48 h and 72 h post inoculation, respectively) as compared to wild-type levels.

### *fumR* is required for fumagillin biosynthesis

**Figure 6:**
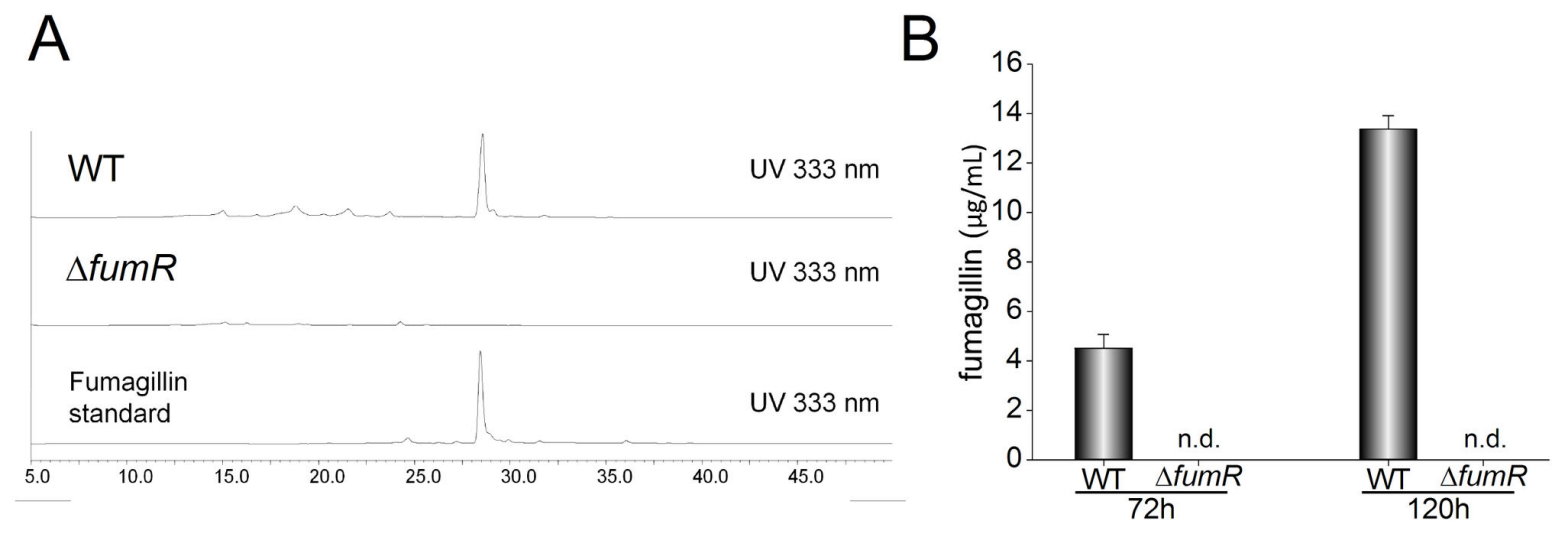
*fumR* regulates fumagillin biosynthesis. *Aspergillus fumigatus* wild type (WT TSD51.1) and ∆*fumR* strains were grown on Czapek-Dox as stationary liquid cultures for 72 h and 120 h. Culture supernatants were extracted with chloroform and analyzed by LC-MS (A) as described in materials and methods. (B) Quantitative data of the LC-MS analysis. The experiment was done with three replicates. Error bars represent standard error. No fumagillin was detected (n.d.) in ∆*fumR* mutants. A standard curve was obtained using commercial fumagillin (Sigma).

Extracts from wild type and ∆*fumR* 72 h and 120 h cultures were subjected to LC-MS analysis as described in the material and methods section. The chemical analysis showed a peak corresponding to fumagillin in the wild type, with a retention time of 29.1 minutes (Figure 6). However, this peak was completely absent in the ∆*fumR* strain, indicating that *A. fumigatus* is unable to produce fumagillin in the absence of *fumR*. The amount of fumagillin production in the wild type increased over time (Figure 6).

### *laeA* regulates expression of *fumR* and the PKS gene Afu8g00370

**Figure 7:**
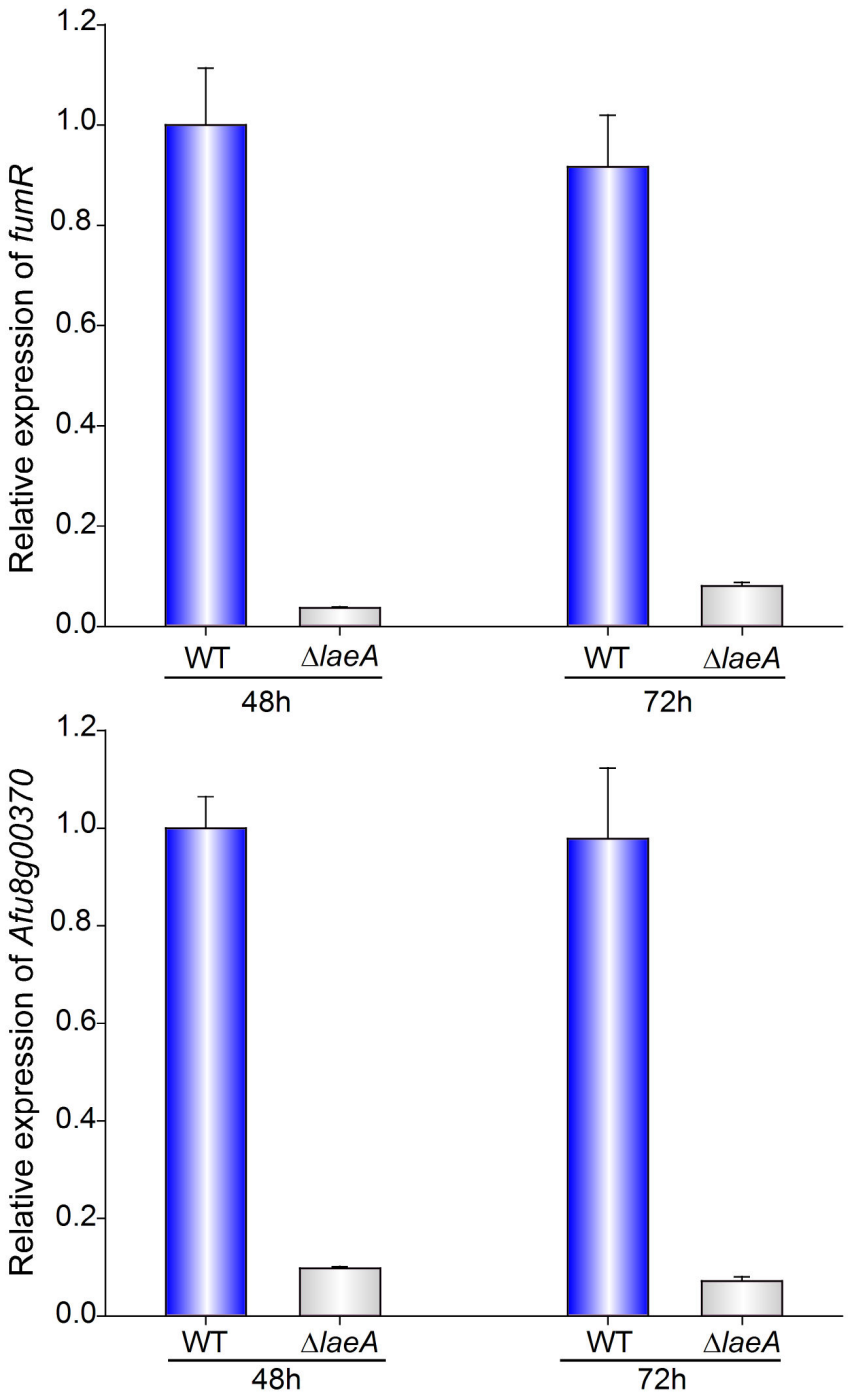
Expression of *fumR* and PKS370 is regulated by *laeA*. Total RNA was extracted from WT and ∆*laeA* strains, grown as stationary liquid Czapek-Dox cultures for 48 h and 72 h. Relative expression of *fumR* and PKS gene Afu8g00370 was calculated using 2^-ΔΔCt^ method as described by Schmittgen and Livak [89]. Primers used for the expression analysis are listed in Table S2. The bar represents the mean of three replicates and error bars represent standard error. Expression of 18S was used as internal reference. Values were normalized to expression levels of wild-type levels considered as 1.

The identity of the cluster involved in fumagillin biosynthesis was not elucidated at the time of the microarray study previously carried out with a ∆*laeA* mutant [51]. In a recent study we described the *A. fumigatus* fumagillin gene cluster [53], and in the present study we have demonstrated that *veA* regulates *fumR*, and that expression of *fumR* is necessary for the activation of other genes in the cluster. We also examined whether the expression of *fumR* was also dependent on *laeA*, analyzing the expression of this gene in a ∆*laeA* mutant and corresponding control strain. The ∆*laeA* strain was constructed as described in material and methods, and the strain was verified by Southern blot analysis (Figure S3). qRT-PCR results indicated a near complete loss of *fumR* expression in ∆*laeA* under conditions that allowed its expression in the control strain (3% and 8% at 48 h and 72 h as compared to wild-type levels) (Figure 7). Expression of Afu8g00370 was also downregulated in Δ*laeA* (9% and 7% at 48 h and 72 h as compared to wild-type levels).

## Discussion

Invasive aspergillosis is a disease caused by the ubiquitous opportunistic invasive mold *Aspergillus fumigatus*. In immunocompromised patients, the intraepithelial immune system of the lung is unable to properly eliminate the inhaled conidia, which then germinate [1–6]. Despite the prevalence of aspergillosis infection and the improvements in diagnosis, novel effective strategies to reduce *Aspergillus* infections are still needed. Deciphering the genetic mechanism controlling *A. fumigatus* cellular processes might provide the basis for the development of new strategies to prevent or treat aspergillosis.

Our study clearly established that *veA* is a global regulator of the *A. fumigatus* genome, affecting the expression of hundreds of genes, many of which are involved in secondary metabolism related processes, in non-random genomic locations. Secondary metabolites, also known as natural products, are part of the fungal chemical arsenal important for habitat adaptation. Some of them are considered virulent factors playing an important role in the pathogen-host interaction. The most studied of these secondary metabolites is gliotoxin, known for its immunosuppressive properties [16,18,72–74], for inhibiting phagocytosis in macrophage, and for induction of apoptosis [29,30]. We recently reported that expression of gliotoxin genes and concomitant gliotoxin production is dependent on *veA* in *A. fumigatus* [13]. In this study we demonstrate that *veA* controls the expression of 14 secondary metabolite gene clusters whose metabolite products are known and an additional 23 putative secondary metabolite gene clusters. Although *veA* exercised negative regulation on some gene clusters, overall *veA* acted as a positive regulator. The *veA*-dependent gene clusters regulatory pattern was not identical to that described for *laeA* [51], which encodes a VeA-interacting protein in the *velvet* complex [31,47,69], presenting some differences in their regulatory output. Among the gene clusters regulated by *veA* are those involved in the synthesis of fumitremorgin G, fumigaclavine C and fumagillin. Production of most of these compounds correlates with the *veA* regulatory pattern observed for the respective gene clusters, with the exception of fumitremorgin, suggesting that in this case other *veA*-dependent factors might be needed for production of this compound at wild-type levels. Fumitremorgin G, fumigaclavine C, and fumagillin, together with production of glionitrin A, currently an orphan compound without an associated gene cluster, have been described to be relevant in the *A. fumigatus* infection process and in other pathologies. Fumitremorgins are associated with dysfunction of the nervous system causing tremors, seizures and abnormal behavior in animals [75,76]. The alkaloid fumigaclavine also causes nervous system damage as well as alteration of the reproductive system [77]. Fumagillin has been associated with invasive aspergillosis due to its effect in slowing ciliary beat frequency [17] and inhibition of endothelial proliferation [17]. Some of these compounds, however, are bioactive molecules with potential or current applications, particularly anti-tumoral compounds such as glionitrin A [78], and the well-known fumagillin and related compounds, with applications against amebiasis [54], microsporidiosis [55], and with known anti-angiogenic activity as inhibitors of the human type 2 methionine aminopeptidase (MetAP2) [56,57]. Our study shows that the expression of most of the genes in the fumagillin gene cluster was negatively affected by either deletion or over-expression of *veA*. This corresponded with a decrease in fumagillin production in these two strains compared to the wild type.

Further insight into the mechanism regulating the secondary metabolite gene cluster in *A. fumigatus* may contribute to decreasing the detrimental effects of this fungus as well as increasing the production of valuable secondary metabolites, such as fumagillin. Our study indicated that the fumagillin gene cluster is regulated by a C6 transcription factor gene, now denominated *fumR*, located within the boundaries of this cluster. Other C6 type transcription factor genes have been found within other secondary metabolite genes clusters, such as the well-known *gliZ* in the *A. fumigatus* gliotoxin gene cluster [18] and *aflR* in the *A. nidulans* sterigmatocystin cluster [79,80], which have been demonstrated to regulate such clusters. *fumR* positively regulated the expression of the recently characterized PKS gene, Afu8g00370, the terpene cyclase gene, Afu8g00520, and the acyltransferase Afu8g00380 [53]. Furthermore, *fumR* also regulates all the other predicted genes in this cluster. Deletion of *fumR* resulted in complete absence of fumagillin production.

Our study showed that *fumR* is under the control of *veA*. Both deletion and over-expression of *veA* downregulated *fumR* transcription, suggesting that *veA* influences the activation of the fumagillin gene cluster through regulation of *fumR*. Over-expression of *veA* also had a marked negative effect on the expression of many *A. fumigatus* secondary metabolite gene clusters. We previously described a similar effect in the *veA* regulation of the gliotoxin gene cluster [13], showing a decrease of gliotoxin production in both deletion and over-expression strains with respect to wild-type levels. Our RNA sequencing data provide strong evidence supporting this pattern. The same pattern has also been observed in other *Aspergillus* species; for example, the production of penicillin has been demonstrated to be negatively affected by either deletion or over-expression of *veA* in *A. nidulans* [37,81]. Since VeA is part of a protein complex or complexes [31,45,47,50,69], we hypothesized that a balanced stoichiometry between VeA and other possible partners might be necessary for proper function, including the activation of secondary metabolite gene clusters. It is possible that other VeA-interacting proteins might also modulate the expression of the fumagillin gene cluster. In this current study we also examined whether *laeA* affects the expression of genes in this cluster. Our results revealed that this is indeed the case; the absence of *laeA* greatly decreases *fumR* and Afu8g00370 expression. This indicates that both VeA and LaeA, components of the fungal *velvet* protein complex, are indispensable for normal expression of the fumagillin gene cluster and fumagillin production in the opportunistic pathogen *A. fumigatus*.

## Conclusion

In this study we have demonstrated that *veA* is a global genetic regulator in the opportunistic human pathogen *A. fumigatus*, controlling the expression of hundreds of genes. Among the genes governed by *veA* are numerous secondary metabolite gene clusters, some of them responsible for the synthesis of natural products considered to be virulent factors during *A. fumigatus* infection. Interestingly, we also showed that some of these *veA*-dependent gene clusters are associated with the production of important medical drugs, such as fumagillin, known for its anti-angiogenic properties among other relevant medical applications. All the genes in the fumagillin gene cluster are under the control of the endogenous regulator *fumR*, which is regulated by *veA* and *laeA*, both encoding interacting components in the *velvet* complex. The findings presented here provide further insight into the regulatory dynamics of the *A. fumigatus* genome, contributing to settinga basis for novel strategies to decrease the negative effects of *A. fumigatus* while increasing its potential to produce beneficial compounds.

## Supporting Information

Figure S1 **qRT-PCR validation of RNA sequencing analysis of the expression of *fumR* (A) and Afu8g00370 (B) in the wild type, ∆*veA*, complementation and OE*veA*.** Total RNA was extracted using TRIzol from 72h old stationary cultures grown in Czapek-Dox medium. The relative expression was calculated using 2^-ΔΔCt^ method as described by Schmittgen and Livak [89]. Primers used for expression analysis are listed in Table S2. The bar represents the mean of three replicates and error bars represent standard error. Expression of 18S was used as internal reference gene. Values were normalized to the expression levels of WT which was considered as 1.
(TIF)

Figure S2 **Targeted *fumR* deletion.** (A) Diagram showing *Sal*I sites (S) in the wild-type *fumR* locus, and the same locus after gene replacement of *fumR* by the *A. parasiticus* *pyrG* gene used as selection marker for fungal transformation. The fragment used as probe templates for Southern blot analyses is also shown. (B) Southern blot analysis. The ∆*fumR* deletion construct was transformed in CEA17ku80 (Table S1). Additional transformants also presented the correct band pattern (data not shown).
(TIF)

Figure S3 **Targeted *laeA* deletion.** (A) Diagram showing *Xho*I sites (X) in the wild-type *laeA* locus, and the same locus after gene replacement of *laeA* by the *A. parasiticus* *pyrG* gene used as selection marker for fungal transformation. The fragment used as probe templates for Southern blot analyses is also shown. (B) Southern blot analysis. The ∆*laeA* deletion construct was transformed in CEA17ku80 (Table S1). Additional transformants also presented the correct band pattern (data not shown).
(TIF)

Table S1 (DOC)

Table S2 (DOC)

Table S3 (XLSX)
