# Supplementary figures and images for: The Fumagillin Gene Cluster, an Example of Hundreds of Genes under veA Control in Aspergillus fumigatus
Source: PLoS One. 2013 Oct 7;8(10):e77147. doi: 10.1371/journal.pone.0077147 (PMC3792039; doi:10.1371/journal.pone.0077147)

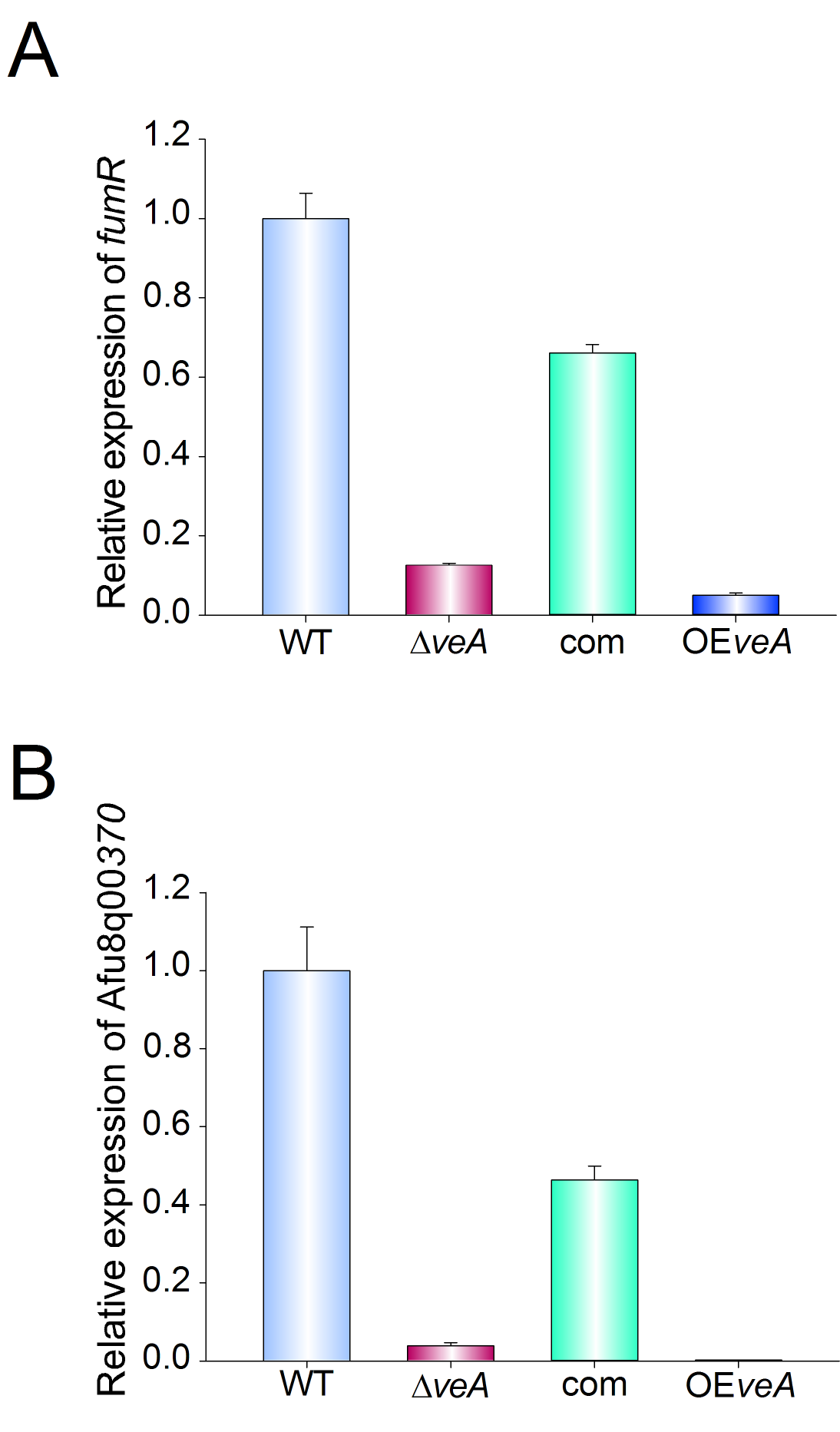

Supplement: Figure S1 — qRT-PCR validation of RNA sequencing analysis of the expression of fumR (A) and Afu8g00370 (B) in the wild type, ∆veA, complementation and OEveA. Total RNA was extracted using TRIzol from 72h old stationary cultures grown in Czapek-Dox medium. The relative expression was calculated using 2-ΔΔCt method as described by Schmittgen and Livak [89]. Primers used for expression analysis are listed in Table S2. The bar represents the mean of three replicates and error bars represent standard error. Expression of 18S was used as internal reference gene. Values were normalized to the expression levels of WT which was considered as 1. (TIF) [file pone.0077147.s001.tif]

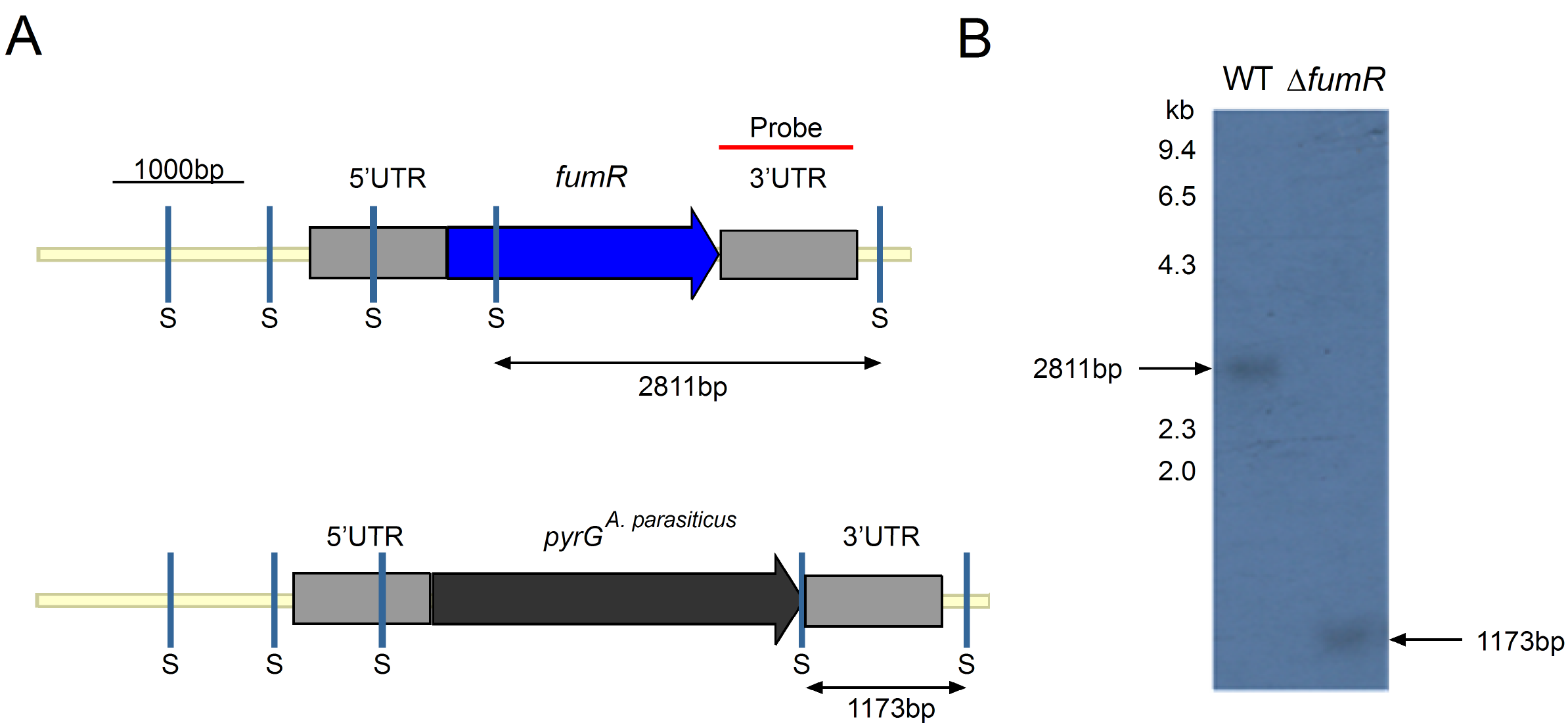

Supplement: Figure S2 — Targeted fumR deletion. (A) Diagram showing SalI sites (S) in the wild-type fumR locus, and the same locus after gene replacement of fumR by the A. parasiticus pyrG gene used as selection marker for fungal transformation. The fragment used as probe templates for Southern blot analyses is also shown. (B) Southern blot analysis. The ∆fumR deletion construct was transformed in CEA17ku80 (Table S1). Additional transformants also presented the correct band pattern (data not shown). (TIF) [file pone.0077147.s002.tif]

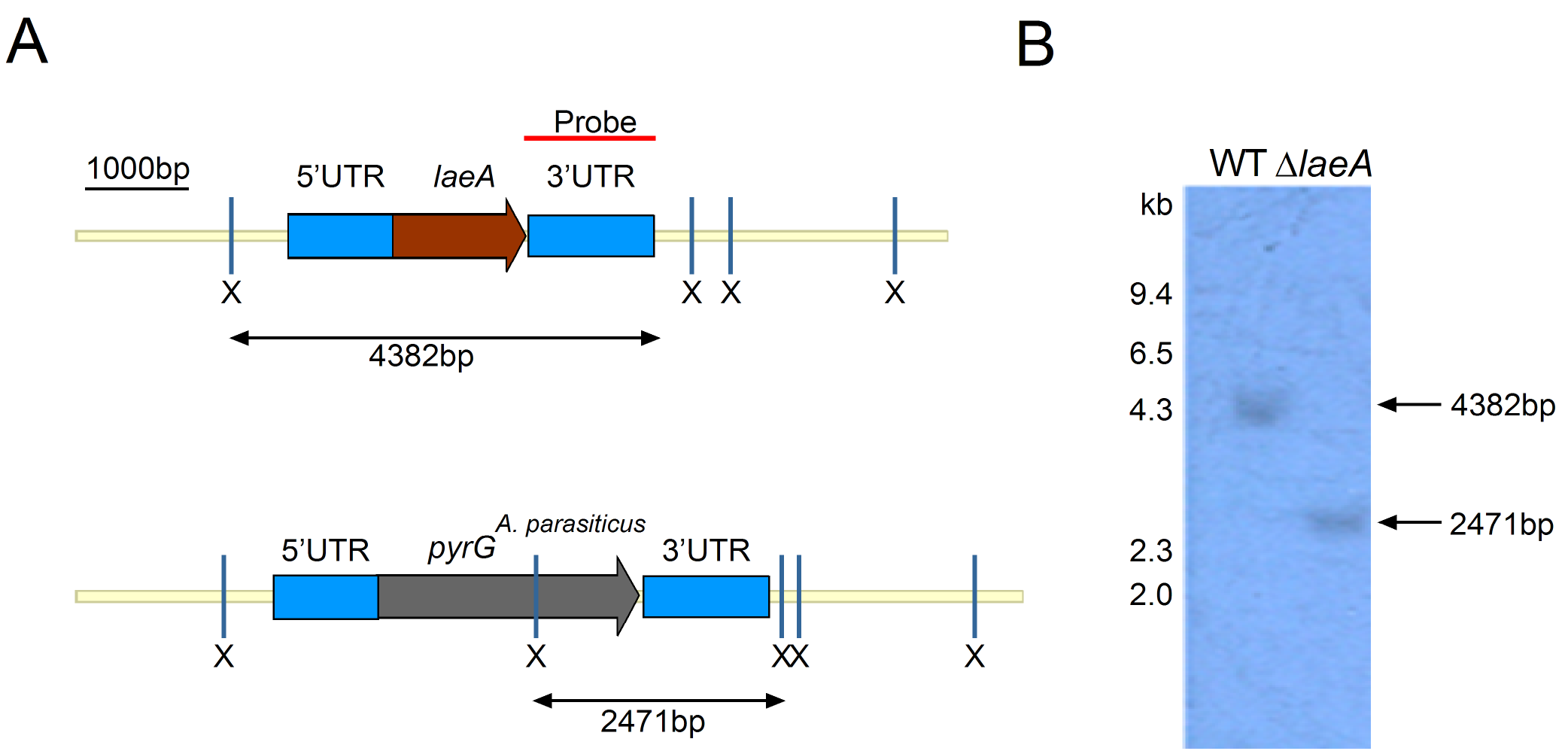

Supplement: Figure S3 — Targeted laeA deletion. (A) Diagram showing XhoI sites (X) in the wild-type laeA locus, and the same locus after gene replacement of laeA by the A. parasiticus pyrG gene used as selection marker for fungal transformation. The fragment used as probe templates for Southern blot analyses is also shown. (B) Southern blot analysis. The ∆laeA deletion construct was transformed in CEA17ku80 (Table S1). Additional transformants also presented the correct band pattern (data not shown). (TIF) [file pone.0077147.s003.tif]
