# Supplementary material for: The Fumagillin Gene Cluster, an Example of Hundreds of Genes under veA Control in Aspergillus fumigatus
Source: PLoS One. 2013 Oct 7;8(10):e77147. doi: 10.1371/journal.pone.0077147 (PMC3792039; doi:10.1371/journal.pone.0077147)
Supplement: Table S1 — (DOC) [file pone.0077147.s004.doc]

**Table S1. Strains used in this study**.

| **Name** | **Genotype** | **Source** |
| --- | --- | --- |
| CEA10 | Wild type | Gift from Robert Cramer |
| TSD1.15 | *pyrG1*; Δ*veA*::*pyrG^A. fumigatus^* | Dhingra et al., 2012 |
| TSD3.5 | *pyrG1*; Δ*veA*::*pyrG*, *veA*::*hyg* | Dhingra et al., 2012 |
| TSD2.8 | *pyrG1*; *gpdA*(p)::*veA*::*trpC*(t)::*pyrG^A. fumigatus^* | Dhingra et al., 2012 |
| CEA17ku80 | *akuB*^ku80^; *pyrG1* | Gift from Robert Cramer |
| TSD51.1 | *akuB*^ku80^; *pyrG1*, *pyrG* *^A. parasiticus^* | This study |
| TSD62.1 | *akuB*^ku80^; *pyrG*; Δ*laeA*::*pyrG^A. parasiticus^* | This study |
| TSD53.1 | *akuB*^ku80^; *pyrG1*; Δ*fumR*:: *pyrG* *^A. parasiticus^* | This study |
