# Supplementary material for: The Fumagillin Gene Cluster, an Example of Hundreds of Genes under veA Control in Aspergillus fumigatus
Source: PLoS One. 2013 Oct 7;8(10):e77147. doi: 10.1371/journal.pone.0077147 (PMC3792039; doi:10.1371/journal.pone.0077147)
Supplement: Table S2 — (DOC) [file pone.0077147.s005.doc]

**Table S**2. Primers used in this study.

| **Name** | **Primer (5’ 🡪 3’)** |
| --- | --- |
| 420p1 | GTCCGAGCCGTCGTCAACTTCCTTTCCAG |
| 420p2 | TCCTACTCTTATCTCCCCAGAGGACCAATTGGCTG |
| 420p3 | ACTGTCAACAGTAGGGGTCCTGTTGTGGTCTTTGC |
| 420p4 | TGGCGTTGACGAGGGGAGAACAGAGAGC |
| 420p5 | CAGCCAATTGGTCCTCTGGGGAGATAAGAGTAGGAACCGGTCGCCTCAAACAATGCTCT |
| 420p6 | GCAAAGACCACAACAGGACCCCTACTGTTGACAGTGTCTGAGAGGAGGCACTGATGCG |
| 420P7 | TCTAGCAAGGGAGGACACCAGGAGAAAAGGGAC |
| 420P8 | GCCGTCAATCGTAGGCGTACCCTGGATATCTGAT |
| laeA_p1 | gacaatcagccatcggagaccctg |
| laeA-p2 | caggtcgaggaggtccaatcgg |
| laeA_p3 | cgccatgaatgcggatctccg |
| laeA_p4 | ctcggcgttgtcgaatggcacc |
| laeA_p5 | ccgattggacctcctcgacctgaccggtcgcctcaaacaatgctct |
| laeA_p6 | cggagatccgcattcatggcggtctgagaggaggcactgatgcg |
| laeA_p7 | ggcaggttggtaccttgctccagag |
| laeA_p8 | gtcctcctggacatttctgacagcg |
| 380qRT_PCR_F | GCTACTCCTTCAGTGCCATGACGG |
| 380qRT_PCR_R | TAGCCGTCATCCCCGACAAACG |
| 430qRT_PCR_F | ACGCTCAAGTCGAGTTCTTCTTCCG |
| 430qRT_PCR_R | CAACCCAGCCGAGGCTGATTTC |
| 440qRT_PCR_F | TTCAACACCGAAGTCAAGTCAGCCC |
| 440qRT_PCR_R | GGCTCCTTGGGAATCGAGGTGATAC |
| 470qRT_PCR_F | TACGCCGCCGTCCCATGTGTAA |
| 470qRT_PCR_R | CCATCTTTGCCTGGGATCTGCG |
| 480qRT_PCR_F | AAAGAGCATGGCTGGGTCAAGATCC |
| 480qRT_PCR_R | GTCCAACTCGGGGAGATAGAAGACG |
| 500qRT_PCR_F | GGGTCAAGAAGGGTGATGTGGTCA |
| 500qRT_PCR_R | TATTCCCCACGATCCCGCACGA |
| 510qRT_PCR_F | CGAGAAGCACATGATCAAGTCGGAGG |
| 510qRT_PCR_R | CAACCTCGGGGAACATGACAAGAC |
| 520qRT_PCR_F | GCCGCAAAACAATCCCGCTAC |
| 520qRT_PCR_R | CAGACAGCGGAGACTCGCAG |
| AparapyrGF-Linker | ACCGGTCGCCTCAAACAATGCTCTGGATCCTATGGATCTCAGAACAATATACC |
| AparapyrGR-Linker | GTCTGAGAGGAGGCACTGATGCGGTCGACATCACCCTTACCCAAACTA |
